# 三种人多发性骨髓瘤细胞系移植小鼠模型的建立和比较

**DOI:** 10.3760/cma.j.issn.0253-2727.2022.05.011

**Published:** 2022-05

**Authors:** 兰婷 刘, 晓晶 魏, 莉欣 龚, 珍 于, 录贵 邱, 牧 郝

**Affiliations:** 中国医学科学院血液病医院（中国医学科学院血液学研究所），实验血液学国家重点实验室，国家血液系统疾病临床医学研究中心，天津 300020 State Key Laboratory of Experimental Hematology, National Clinical Research Center for Blood Diseases, Institute of Hematology & Blood Diseases Hospital, Chinese Academy of Medical Sciences & Peking Union Medical College, Tianjin 300020, China

**Keywords:** 多发性骨髓瘤, 模型, 小鼠, Multiple myeloma, Models, Mice

## Abstract

**目的:**

利用人骨髓瘤细胞系ARP1、MM.1S和NCI-H929建立异种移植模型，并对三种细胞移植后生长周期、肿瘤负荷和生物学特点进行比较。

**方法:**

将ARP1、MM.1S和NCI-H929细胞分别由皮下或尾静脉植入经^137^Cs照射后的NOD/SCID小鼠，每周观察小鼠的生存情况，监测肿瘤负荷。应用流式细胞术检测小鼠肿瘤组织或骨髓中CD138^+^细胞的比例。采用免疫荧光检测肿瘤组织细胞的CD138和免疫球蛋白轻链表达。ELISA法检测骨髓和外周血中的免疫球蛋白轻链，micro-CT评价骨病变。

**结果:**

皮下移植ARP1、MM.1S和NCI-H929细胞的小鼠在两周内均可形成局部肿瘤，免疫荧光检测支持浆细胞肿瘤。尾静脉移植后第20天时可在ARP1组小鼠外周血中检测出κ轻链［（8.2±1.0）ng/ml］。尾静脉移植6周左右，ARP1组小鼠出现体重下降、精神萎靡、下肢逐渐瘫痪等表现，骨髓中能检测到人CD138^+^CD38^+^细胞群，予硼替佐米治疗可显著降低肿瘤负荷［（5.7±0.2）％对（21.3±2.1）％，*P*<0.01］。MM.1S和NCI-H929组小鼠骨髓中未检测到人CD138^+^CD38^+^细胞群。

**结论:**

ARP1、MM.1S和NCI-H929细胞系构建的小鼠模型可作为MM发病机制及临床研究的良好模型。

## Abstract (en)

**Objective:**

To establish three types of xenotransplantation models using human myeloma cell lines ARP1, MM.1S, and NCI-H929 and to compare the proliferation, tumor load, and biological characteristics of the three types of cells after transplantation.

**Methods:**

Suspensions of human myeloma cell lines ARP1, MM.1S, and NCI-H929 were implanted into NOD/SCID mice by subcutaneous injection or tail vein injection. The survival of the mice was observed weekly, and the tumor load was measured. Flow cytometry was used to detect the proportion of CD138^+^ cells in tumor tissue or the mouse bone marrow. CD138^+^ cells and light chains were detected by immunofluorescence. Light chains in bone marow and peipheral blood were measured by ELISA, and bone disease was assessed by micro-CT.

**Results:**

Mice injected with ARP1, MM.1S, and NCI-H929 cells all formed tumors subcutaneously in about 2 weeks. Immunofluorescence detection supported plasma cell tumors. Kappa light chains were detected in the peripheral blood of ARP1 mice on day 20 after tail vein transplantation（8.2±1.0 ng/ml）. After 6 weeks of tail vein transplantation, mice in the ARP1 group showed signs of weight loss, mental depression, and dragging legs, and human CD138^+^CD38^+^ cells were detected in the bone marrow（BM）. Furthermore, bortezomib（BTZ）treatment given once the tumor was established significantly reduced the tumor burden［（5.7±0.2）％ vs（21.3±2.1）％，*P*<0.01］. Human CD138^+^CD38^+^ cells were not detected in the BM of the MM.1S or NCI-H929 groups.

**Conclusion:**

The results of this study suggest that the mouse models constructed by the three cell lines（ARP1, MM.1S, and NCI-H929）can be used as models for the pathogenesis and clinical research of MM.

多发性骨髓瘤（MM）是一种恶性浆细胞肿瘤，其特征是浆细胞在骨髓微环境中克隆性增殖并产生大量单克隆免疫球蛋白（Ig）[1]。虽然近年来新药的使用延长并改善了患者的生存情况，但耐药的发生导致骨髓瘤仍然是一种不可治愈的疾病。骨髓瘤动物模型的建立为进一步探索耐药机制和开发新的治疗方案提供了强有力的支持，各种满足实验需求的小鼠模型应运而生，其中具有不同生物学特性的骨髓瘤细胞系，如RPMI-8226、U266、ARP1、KMS-12、MM.1S以及NCI-H929等通过不同途径植入免疫缺陷小鼠，是一类较基因改造小鼠或患者原代细胞移植小鼠更为稳定常用的建模方法[2]–[12]。此类模型中局部肿瘤的成瘤率和髓内肿瘤的植入率则取决于细胞数量、种类、移植处理方案以及小鼠类型等。本研究对比了三种具有不同遗传学异常的人骨髓瘤细胞系移植NOD/SCID小鼠建模[13]–[15]，为建立体内和局部骨髓瘤模型提供参考方案。

## 材料与方法

1. NOD/SCID小鼠皮下移植模型：4～5周龄雌性NOD/SCID小鼠购自维通利华实验动物技术有限公司［中国北京，动物质量合格证号：SCXK（京）2016-0006］，体重18～20 g，饲养于中国医学科学院血液学研究所SPF级动物实验室［实验单位使用许可证号：SYXK（津）2020-0003］。NCI-H929和MM.1S两组小鼠移植前24 h接受200 cGy全身照射。人骨髓瘤细胞系ARP1 1.0×10^6^、NCI-H929 1.0×10^7^或MM.1S 5.0×10^6^分别经皮下注射至小鼠，最终体积100 µl/只。使用游标卡尺测量肿瘤最大直径（a）与最小直径（b），按照公式ab^2^/2计算肿瘤体积，当肿瘤体积达到2 000 mm^3^时处死小鼠。

2. NOD/SCID小鼠尾静脉移植模型：移植前24 h予NOD/SCID小鼠250 cGy全身照射。人骨髓瘤细胞系ARP1 2.0×10^6^、NCI-H929 1.0×10^7^或MM.1S 5.0×10^6^分别经尾静脉注射植入小鼠，最终体积为300 µl/只。ARP1组移植3周后，将小鼠随机分为两组，分别给予硼替佐米（BTZ）（1 mg/kg）和磷酸盐缓冲液（PBS），每周2次腹腔注射100 µl。在移植肿瘤细胞6周后对所有小鼠实施安乐死。取一侧股骨和胫骨的骨髓细胞，制备单细胞悬液。另一侧股骨和胫骨用10％中性福尔马林固定。

3. 细胞培养：MM细胞系NCI-H929和MM.1S购自ATCC细胞库，ARP1细胞系由美国小石城阿肯色大学医学院骨髓瘤中心湛凤凰教授馈赠。三种细胞系的遗传学异常见表1。MM细胞系在RPMI 1640培养基中培养，加入10％热灭活胎牛血清（FBS）、青霉素（100 IU/ml）和链霉素（100 µg/ml），置于37 °C、5％CO_2_饱和湿度培养箱中扩增培养，取对数生长期细胞进行实验。

**表1:** FISH检测ARP1、MM.1S、NCI-H929细胞系遗传学异常结果

细胞系	RB1缺失	P53缺失	1q21扩增	IGH易位
ARP1	-	+	+	t（14;16）
MM.1S	+	-	+	t（14;16）
NCI-H929	-	-	-	t（4;14）

4. 流式细胞术：采集骨髓或肿瘤组织样本，制备成单细胞悬液，标记抗人CD138和CD38抗体（BD Biosciences）以确定是否存在人骨髓瘤细胞。在FACS CantoⅡ机器上进行流式细胞术检测，用Flow-Jo软件进行数据分析。

5. 免疫荧光分析：用4％多聚甲醛固定脱钙骨制备冰冻组织切片。对上述小鼠切除的肿瘤组织进行CD138、免疫球蛋白轻链κ（Ig-κ）和λ（Ig-λ）的免疫荧光检测。将载玻片固定在−20 °C甲醇中5 min，置于含有0.05％Tween-20的磷酸盐缓冲溶液中5 min，然后在室温下用含5％牛血清白蛋白的PBS封闭1 h。在室温下与抗体CD138和Ig-κ或Ig-λ（1∶100稀释于含4％牛血清白蛋白的PBS中，英国Abcam公司产品）孵育1 h后，用PBS-T轻轻冲洗载玻片3次（3 min/次），然后在室温下与AlexaFluor488山羊抗鼠和AlexaFluor568山羊抗兔IgG（IgG；H+L；1∶200，美国Invitrogen公司）结合的二抗孵育30 min。使用共聚焦显微镜（美国Perkineller公司）拍摄图像。

6. ELISA检测：以来源于人骨髓瘤细胞分泌的Ig轻链水平作为监测肿瘤负荷的间接指标。每周从小鼠面颊静脉中采集外周血并分离获得血清样本，采用酶联免疫吸附试剂盒（美国Bethyl Laboratories 公司）测定小鼠血清中人Ig轻链。实验结束时，检测骨髓浆和血清中Ig轻链，分析小鼠肿瘤细胞负荷情况。用微量滴定读板仪测定吸光度（450 nm），用CurveExpert 1.4软件计算Ig轻链含量。

7. Micro-CT分析：用10％中性福尔马林固定小鼠股骨和胫骨，并进行micro-CT分析。股骨和胫骨扫描使用量子GX显微成像系统（PerkinElmer）。

8. 统计学处理：使用GraphPad Prism 5.0进行统计学分析。组间分析采用*t*检验，实验结果以均数±标准差表示，*P*<0.05为差异有统计学意义。

## 结果

1. 皮下移植模型：NOD/SCID小鼠左腹部皮下注射骨髓瘤细胞，移植10 d后开始记录肿瘤的大小，每10 d测量一次。MM.1S组小鼠肿瘤生长速度较快，于移植后20 d终止实验，肿瘤平均体积为1 738.3 mm^3^。ARP1组与NCI-H929组在移植后30 d终止实验，两组肿瘤平均体积为1 761.1 mm^3^和1 445.3 mm^3^，生长速度的差异无统计学意义（*P*>0.05）（图1）。流式细胞术检测肿瘤单细胞悬液显示肿瘤组织中存在高比例CD138^+^细胞，三组CD138^+^细胞的比例均超过60％（图2）。同时，肿瘤组织切片进行CD138和Ig-λ或Ig-κ免疫荧光染色（图3），在三组肿瘤组织中均可检测到Ig轻链。

**图1:**
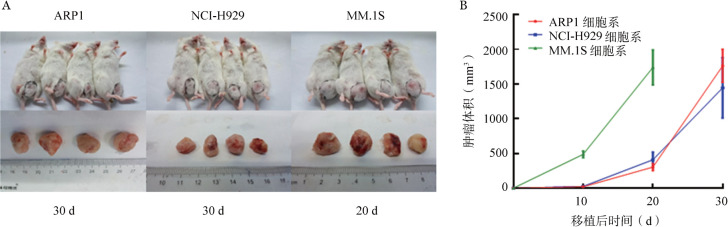
ARP1、NCI-H929或MM.1S细胞皮下移植至NOD/SCID小鼠腹部后肿瘤体积（每组4只小鼠）

**图2:**
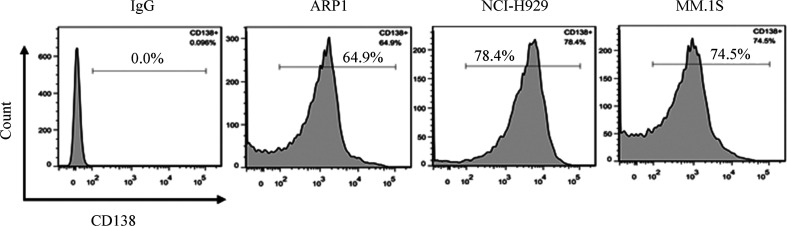
流式细胞术分析肿瘤细胞中CD138^+^细胞比例（每组4只小鼠）

**图3:**
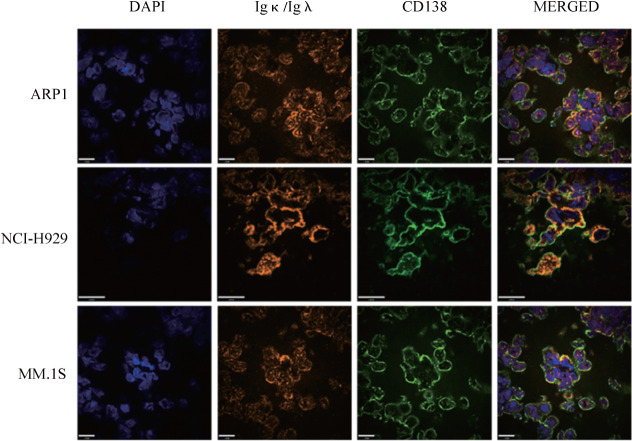
免疫荧光检测肿瘤组织中CD138^+^细胞（绿色）表达Ig λ或Ig κ（橘色）情况

2. 尾静脉移植模型：我们比较了三种细胞系经尾静脉注射至照射后小鼠后的体内归巢能力。移植后每10 d采集小鼠面颊外周血，ELISA法检测血清中人MM细胞分泌轻链水平以监测肿瘤负荷。在移植后第20天ARP1组能检测到κ轻链，随着移植时间延长，κ轻链表达水平明显上升（图4），而其他组小鼠血清中均未能检测到轻链表达。在实验终点，流式细胞术检测发现MM.1S组和NCI-H929组小鼠移植后6周骨髓中仍未检测到明显的CD138^+^CD38^+^细胞群，而ARP1组小鼠骨髓中能检测到较高比例的人CD138^+^CD38^+^细胞［（21.3 ± 2.1）％］，说明经尾静脉注射ARP1细胞可通过血液循环成功归巢到小鼠骨髓腔中（图5）。BTZ是一种临床常用的蛋白酶体抑制剂，可显著改善MM的疗效[16]。注射ARP1细胞3周后，将小鼠随机分为两组（每组3只），分别予BTZ（ARP1-BTZ组）和PBS（ARP1组），每周2次。经过3周的治疗，ARP1-BTZ组小鼠的骨髓微环境中CD138^+^CD38^+^细胞的比例较ARP1组明显降低［（5.7±0.2）％ 对（21.3±2.1）％，*P*<0.01］（图5），说明ARP1细胞对BTZ较为敏感。ARP1组血清κ轻链浓度在移植后3～6周由（8.2±1.0）ng/ml上升到（402.9±73.0）ng/ml，ARP1-BTZ组则由（8.8±0.2）ng/ml缓慢上升到（54.2±18.9）ng/ml，ARP1-BTZ组在第40天时κ轻链浓度明显低于ARP1组（*P*<0.01）（图4）。ARP1-BTZ组骨髓浆中轻链表达水平也较ARP1组显著降低［（6.40±0.50）µg/ml对（0.90±0.04）µg/ml，*P*<0.001］（图4）。在实验终点，ARP1模型中的小鼠出现后肢瘫痪、体重明显减轻等症状。micro-CT仪器检测小鼠腿骨的骨破坏情况。与未移植ARP1的NOD/SCID小鼠（正常对照组）相比，注射ARP1细胞的小鼠后肢出现明显的骨损伤，CT显示骨体积［（7.0±1.2）％对（27.7±5.4）％，*P*<0.05］及骨小梁厚度［（19.7±2.6）％对（50.0±5.8）％，*P*<0.01］均明显降低（图6）。

**图4:**
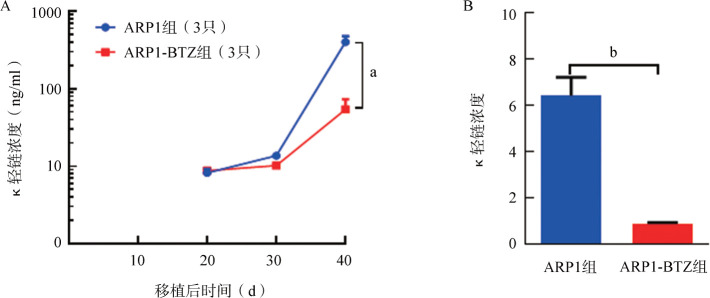
ELISA法检测ARP1组和ARP1-BTZ组小鼠外周血清（A）和移植后40 d骨髓浆（B）中人κ轻链浓度（每组3只小鼠） ^a^*P*<0.01，^b^*P*<0.001

**图5:**
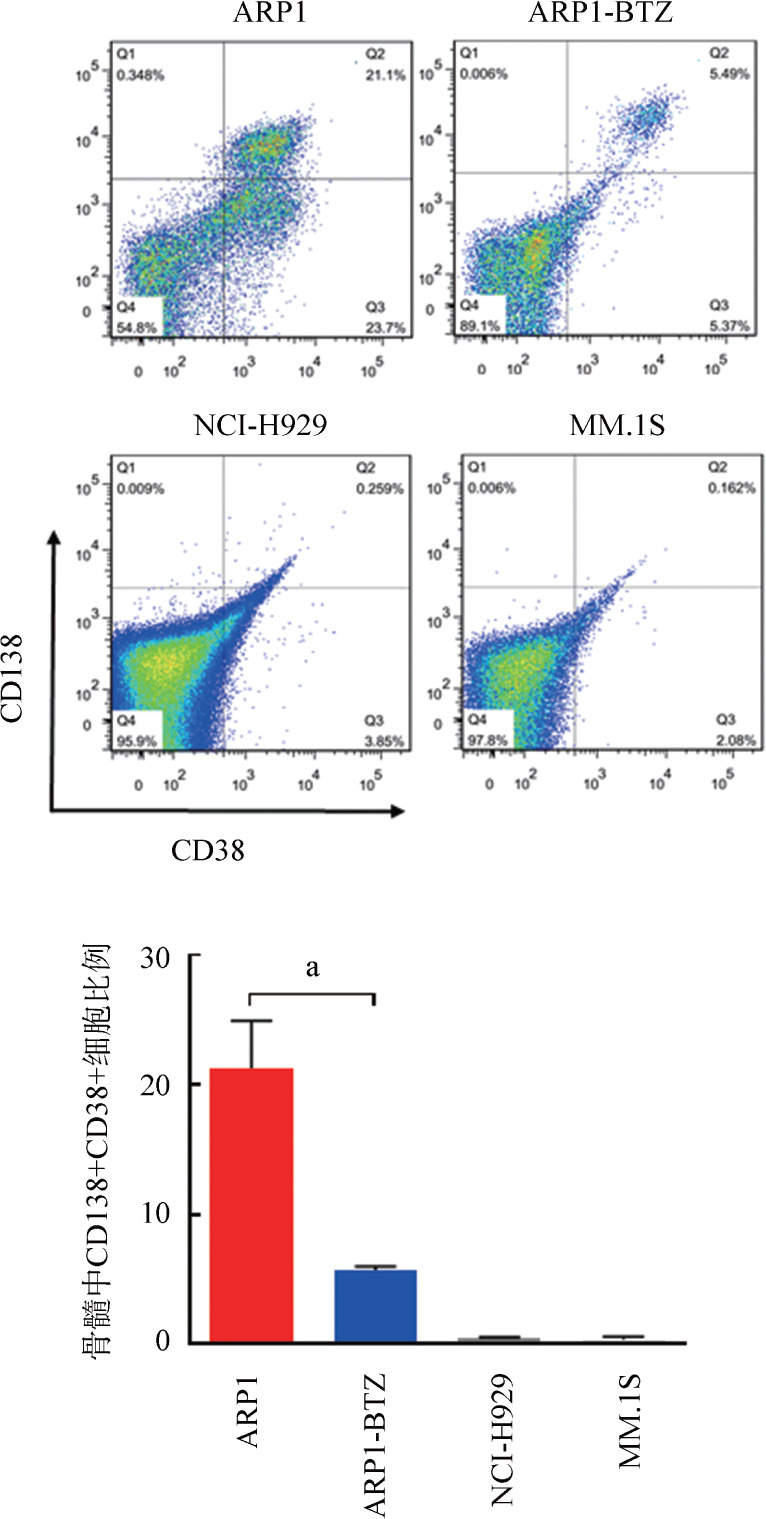
流式细胞术检测移植后6周各组小鼠骨髓中CD138^+^CD38^+^细胞比例（每组3只小鼠） ^a^*P*<0.01

**图6:**
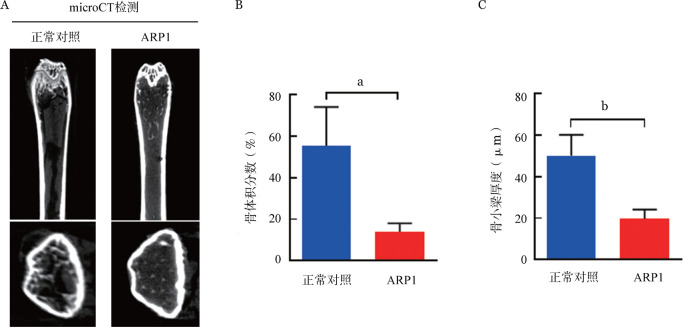
正常对照组和ARP1组小鼠腿骨骨破坏情况（A）、骨体积分数（B）和骨小梁厚度（C）（每组3只小鼠） ^a^*P*<0.05，^b^*P*<0.01

## 讨论

本研究旨在建立稳定、高效、重复性好的MM小鼠模型。三种细胞形成的移植模型有各自的特点，均能在短时间内形成局部肿瘤，可用于评估药物对皮下瘤体生长的作用及肿瘤细胞行基因改造后的生物学变化等。但这类模型不能反映骨髓微环境对肿瘤细胞的影响，也无法观察药物对骨髓瘤并发症如骨损伤、贫血的疗效。而移植后肿瘤细胞归巢定居到骨髓中的ARP1小鼠模型刚好克服了上述皮下移植模型的缺陷，能较好地模拟骨髓瘤的疾病进程，可广泛用来研究骨髓瘤发病机制和新药筛选。

目前用于骨髓瘤移植模型的小鼠包括免疫低下小鼠（如SCID）、免疫缺陷小鼠（如NOD/SCID）和严重免疫缺陷小鼠（如NSG、NOG等）。常用的移植方法有皮下局部移植、静脉移植和髓腔移植等。髓腔移植虽然能精准植入骨髓提高植入效率，但注射细胞过程中容易造成肿瘤细胞渗漏到周围肌肉组织中，影响实验结果。值得注意的是，MM.1S组和NCI-H929组小鼠在皮下移植前需要进行全身半致死剂量照射以确保植入成功率。由于NOD/SCID小鼠的NK细胞尚存活性，大多数骨髓瘤细胞系非照射移植至小鼠后成瘤率明显下降，且成瘤时间差异较大，不利于实验研究。高剂量照射在一定程度上增加了荷瘤小鼠的自然死亡率，对观察药物疗效的实验影响较大。有研究证明，300 Rads的亚致死剂量全身照射后的NOD/SCID小鼠需植入3.0×10^6^～1.0×10^7^个MM.1S细胞才能造模成功[17]。近年来，Shancer等[18]在NSG小鼠体内注射1×10^7^个H929细胞，成功建立了肿瘤细胞骨髓归巢小鼠模型。还有实验室仅用0.5×10^6^个ARP1细胞尾静脉注射至NOD.Cγ-Rag1小鼠即能使肿瘤细胞全身扩散[4]。因此MM.1S和NCI-H929细胞可能需要严重免疫缺陷小鼠作为受体，使用更多数量细胞移植或更大剂量的辐照清髓建立体内模型。

综上所述，本研究成功地建立了两类MM小鼠模型。皮下移植模型可局部注射形成浆细胞实体肿瘤。该模型具有成瘤率高、操作简单、肿瘤生长观察直观等优点，可作为实验研究的辅助模型。与皮下移植模型相比，尾静脉注射ARP1细胞小鼠模型能更好地反映MM细胞与骨髓微环境之间的相互作用，表现出骨髓瘤患者典型的临床特征，为探索MM发病机制和治疗靶点提供合适的动物模型。
